# Left Atrial Posterior Wall Thrombus After Posterior Wall Ablation

**DOI:** 10.31486/toj.18.0148

**Published:** 2020

**Authors:** Shourjo Chakravorty, Sangeeta Shah, Michael L. Bernard

**Affiliations:** ^1^ The University of Queensland Faculty of Medicine, Ochsner Clinical School, New Orleans, LA; ^2^ Department of Cardiology, Ochsner Clinic Foundation, New Orleans, LA

**Keywords:** *Ablation techniques*, *atrial fibrillation*, *coronary thrombosis*, *heart atria*

## Abstract

**Background:** Posterior wall isolation for recurrent atrial arrhythmia is a commonly used technique to achieve long-term freedom from atrial fibrillation. Despite the widespread use of posterior wall isolation, its long-term effects on left atrial function are unknown. Specifically, the effect of isolated atrial walls on stasis and risk of thrombus has not been established. We present the case of a patient who developed a left atrial posterior wall thrombus after a posterior wall isolation attempt.

**Case Report:** A 67-year-old female with a complex electrophysiologic history was found to have a left atrial posterior wall thrombus when she presented for a third ablation attempt for drug-refractory macroreentrant left atrial tachycardia 5 weeks after a posterior wall isolation attempt. The patient had a number of risk factors that could have been associated with the unusually located thrombus: hypertension, low ejection fraction, mitral valve disease, and recurrence and sustained duration of symptomatic atrial fibrillation. After the patient had 3 weeks of anticoagulation treatment, transesophageal echocardiography showed no left atrial thrombus, and she underwent successful reisolation of the posterior wall. The third ablation was successful, and the patient developed no complications of stroke, transient ischemic attack, or systemic embolization throughout her treatment course.

**Conclusion:** To our knowledge, this case is the second report of a left atrial posterior wall thrombus in this setting. The patient's complex and specific set of risk factors likely led to this rare finding. Although left atrial posterior wall thrombus after ablation is rare, in patients with specific risks or a combination of factors that could lead to such a clot, visualizing the left atrium in these patients may be beneficial to minimize the risk of systemic embolization.

## INTRODUCTION

Radiofrequency, cryoablation, and surgical ablation have vastly improved the outcomes of atrial fibrillation (AF) management compared with anticoagulation therapy.^1^ Although arrhythmias recur after initial ablation attempts, repeat procedures are often successful.^2^ Known causes for AF recurrence include incomplete pulmonary vein isolation (PVI), extrapulmonary vein triggers, and macroreentrant circuits generated from PVI.^3^ For patients who are refractory to one or more antiarrhythmic agents or do not want to take such medications, radiofrequency ablation (RFA) therapy has become the standard of care.^3^ Although the success rate for a single PVI for longstanding AF is 50% to 70% and recurrence rates are 20% to 30%, the procedure is still the best modality for long-term treatment for symptomatic patients.^3^

**Figure 1.**
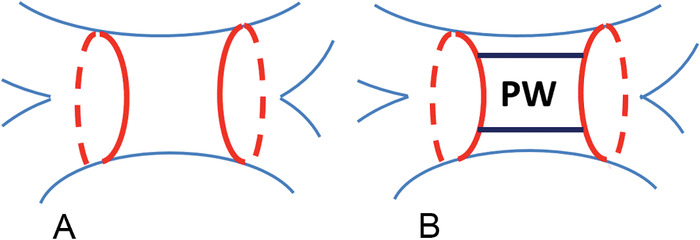
**Schematic of left atrial posterior wall (PW) isolation lesion set. After completion of wide area circumferential ablation set (A), superior and inferior PW linear lesion sets are made to isolate the PW of the left atrium (B).**

In the procedure, linear ablation connects the superior and inferior portions of the posterior wide area circumferential ablation lines (Figure 1), creating a continuous zone of electrical isolation from one set of pulmonary veins across the posterior wall to the pulmonary veins on the opposite side. Incomplete isolation of the posterior wall can result in reentrant left atrial arrhythmias. A common approach for those who fail an initial PVI procedure is to isolate the posterior left atrium during subsequent procedures, thus allowing for the ablation of nonpulmonary vein sources.^4^

Isolation along the posterior wall carries unique risks relative to the remainder of the left atrium. Most prominently, the posterior wall is proximate to the esophagus, thus requiring meticulous attention to esophageal heating. Also, the relative thinness of the posterior wall can increase left atrium perforation risk. Other known risks include maintained conductance of AF as a result of incomplete linear ablation sets.^5^ Another complication of cardiac RFAs is bleeding with cardiac tamponade, a rare but potentially life-threatening complication.^6^ Other complications include the development of an atrioesophageal fistula, torsades de pointes, pulmonary vein occlusion, tracheal compression secondary to subclavian hematoma, and acute respiratory distress syndrome.^7^

We present the case of a patient who developed a left atrial posterior wall thrombus after a posterior wall isolation attempt.

## CASE REPORT

**Figure 2.**
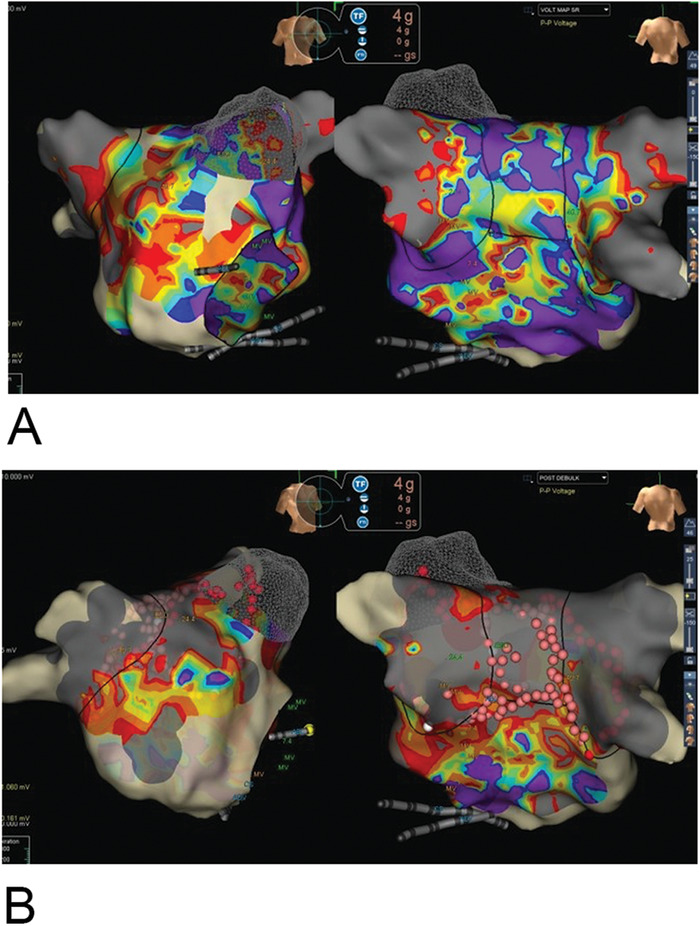
**Electroanatomic voltage map of the left atrium before (A) and after (B) posterior wall isolation. Isolation of the posterior wall is evidenced by the homogenous gray area spanning the pulmonary veins and posterior wall. Ablation lesions are marked by the dots in view B.** Note: A color version of this graphic is available at www.ochsnerjournal.org.

**Figure 3.**
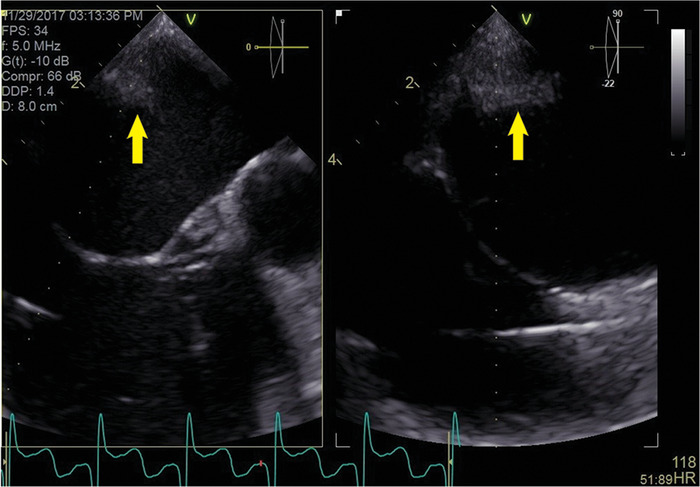
**Transesophageal echocardiogram shows 15 × 11 mm posterior wall thrombus (arrows).**

**Figure 4.**
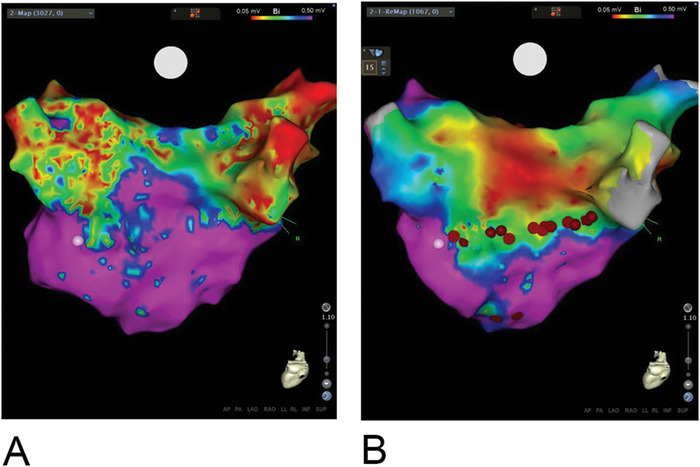
**Electroanatomic voltage map of the left atrium before (A) and after (B) repeat posterior wall isolation. Compared with the first ablation, there was reconnection along the inferior posterior wall lesion set. The light/dark interface marks the boundary of isolated (light) and active (dark) tissue. Red represents areas of electrical isolation.** Note: A color version of this graphic is available at www.ochsnerjournal.org.

A 67-year-old female with a history of hypertension, arrhythmia-induced cardiomyopathy, heart failure with ejection fraction (EF) of 15%, AF treated with 5 mg twice daily with apixaban, sick sinus syndrome (SSS), and mitral regurgitation presented for her third ablation attempt for drug-refractory macroreentrant left atrial tachycardia. Her first ablation, using a cryoballoon, had been performed 21 months prior to this presentation. She was instructed to not take apixaban for 24 hours prior to the ablation. After the cryoballoon procedure, the patient required cardioversion for recurrence of AF. Amiodarone was used for a short period but was stopped because of side effects. The patient maintained normal sinus rhythm with normalization of EF for 11 months but then experienced paroxysmal AF recurrence. She developed tachycardia-bradycardia syndrome that required a dual chamber pacemaker implantation. She progressed into persistent AF, and her EF decreased to 35%. RFA was performed. Her pulmonary veins were isolated from the initial ablation procedure (Figure 2A), and a posterior box lesion set was performed (Figure 2B). Three weeks after the RFA, the patient developed a left atrial macroreentrant arrhythmia with worsening heart failure (EF of 15%). Transesophageal echocardiogram (TEE) at 5 weeks showed a posterior left atrial thrombus that precluded a third ablation attempt (Figure 3). Again, she had held apixaban for less than 24 hours prior to her ablation procedure. Apixaban was increased to 10 mg twice daily for 3 weeks before a third ablation was attempted. TEE at this time showed no left atrial thrombus. The patient underwent successful reisolation of the posterior wall. The inferior portion of her posterior wall was active with conduction block along the roof (Figures 4A and 4B). The third ablation was successful; sinus rhythm and normal EF were restored. The patient developed no complications of stroke, transient ischemic attack, or systemic embolization throughout the entire treatment course.

## DISCUSSION

The posterior wall of the left atrium provides a substrate for the perpetuation of persistent AF. Electrical isolation of the posterior wall for patients with persistent AF after initial RFA is a management option.^8^ We suspect that the posterior wall isolation approach may have an underrecognized risk of formation of a posterior wall clot in select patients with prior left atrial dysfunction. While thrombus was correctly identified in this case, another differential to consider for a left atrial mass is a cardiac myxoma.^9^ Myxomas tend to be solitary, pedunculated, and mobile. They are often difficult to differentiate from a thrombus. A trial of anticoagulation can help differentiate the two, as this pharmacologic treatment will not resolve a myxoma.^9^

This case highlights a confluence of factors that may have contributed to the formation of this unusually located thrombus. The literature suggests that up to 90% of AF-induced clots form in the left atrial appendage.^10^ Enlargement of the cardiac chambers causes stasis of blood and an increased risk of thrombus formation in the left atrial appendage and the left atrium itself.^11^ Our patient, with a history of cardiomyopathy and mitral regurgitation, had this risk. One paper suggests that chronic mitral regurgitation can cause a rare type C calcification in the posterior wall of the left atrium.^12^ Calcification would increase clot formation risk at this location specifically. Other papers suggest that a low EF is an independent major risk factor for thrombus formation.^13,14^ The patient in our case had an EF as low as 10% to 15% during her course of treatment. To our knowledge, our case is the second report of a left atrial posterior wall thrombus in this setting.^15^

In a case similar to ours, a 77-year-old female underwent a maze procedure for management of paroxysmal AF with aortic valve disease and eventually underwent pacemaker implantation for SSS. Two years postoperatively, a large thrombus was found in the patient's posterior left atrium.^16^ This chronology of cardiac diagnoses and management is similar to our case. In another case, a 66-year-old female was found to have a left atrial thrombus after undergoing mitral valvuloplasty and maze procedure for mitral regurgitation and atrial fibrillation.^17^ This case suggested the importance of anticoagulation therapy for those undergoing mitral valvuloplasty despite maintaining sinus rhythm. The formation of left atrial clots is increased after performing maze procedure due to damage being caused to the wall, especially in patients with preexisting mitral valve conditions.^17^ Additionally, it is important to note that thrombi that form after RFA are often missed despite intraprocedural anticoagulation because postablation imaging is usually not performed.^18^ One proposed mechanism for the resulting thrombus is due to the sudden rise in temperature upon impedance of the ablation electrode. This event has been shown to occur with electrode temperatures near 100°C, thus causing protein denaturation and aggregation.^19^

Our case highlights 3 important considerations for clinicians. First, the formation of a thrombus can be a complication of performing the actual isolation procedure itself. Second, the posterior wall of the left atrium is an unusual location for a thrombus to form, so it is usually missed on the majority of echocardiograms that focus on the left atrial appendage. Third, operators who perform ablations should be aware of the possible risk of posterior wall clot formation during an ablation that carries an unknown risk of stroke and systemic embolism (Table).

**Table.** Risk Factors and Pathophysiology for Cardiac Thrombosis

Risk Factor	Pathophysiology
Mitral valve disease (prolapse, stenosis, regurgitation)	Enlargement of the left atrial chamber/stasis
Chronic heart failure low ejection fraction	Decreased cardiac output→stasis
Duration of atrial fibrillation/Recurrence of atrial fibrillation	Increased thrombosis risk
History of radiofrequency ablation	Damaged wall acts as nidus for clot formation

## CONCLUSION

A complex combination of risk factors—hypertension, low EF, mitral valve disease, recurrence and sustained duration of symptomatic AF—triggered the formation of a posterior wall clot in our patient. Although such cases are rare, recognizing that an isolation procedure or maze procedure may contribute to the formation of such thrombi is important. Also important to consider is what specific risks or combination of factors may lead to an abnormally located clot; visualizing the left atrium in select patients with these risk factors may be beneficial to minimize the risk of systemic embolization.

## Notes

This article meets the Accreditation Council for Graduate Medical Education and the American Board of Medical Specialties Maintenance of Certification competencies for Patient Care and Medical Knowledge.
